# The Cognitive and Neuroimaging for Neurodegenerative Disorders Study (CogNID): design and initial findings from real-world clinical practice

**DOI:** 10.3389/fpsyt.2025.1630082

**Published:** 2025-10-29

**Authors:** Akram A. Hosseini, Beili Shao, Abigail Rebecca Lee, Permesh Dhillon, Kehinde Junaid, Bruno Gran, Peter Sellars, Hannah Sargisson, JeYoung Jung, Elizabeta B. Mukaetova-Ladinska

**Affiliations:** ^1^ Department of Neurology, Nottingham University Hospitals NHS Trust, Nottingham, United Kingdom; ^2^ Sir Peter Mansfield Image Centre, University of Nottingham, Nottingham, United Kingdom; ^3^ Academic Neurology, Mental Health and Clinical Neuroscience Academic Unit, University of Nottingham School of Medicine, Nottingham, United Kingdom; ^4^ Radiological Sciences, Mental Health and Clinical Neuroscience, School of Medicine, University of Nottingham, Nottingham, United Kingdom; ^5^ Old Age Psychiatry, Nottinghamshire Healthcare NHS Foundation Trust, Nottingham, United Kingdom; ^6^ Department of Psychology, University of Nottingham, Nottingham, United Kingdom; ^7^ School of Psychology and Visual Sciences, University of Leicester, Leicester, United Kingdom; ^8^ The Evington Centre, Leicester General Hospital, Leicester, United Kingdom

**Keywords:** cognitive impairment, dementia, cohort, neuroimaging, neurodegenerative disorders, mild cognitive impairment, early onset dementia, memory clinic

## Abstract

**Introduction:**

Dementia presents with significant heterogeneity across age groups, particularly in early-onset cognitive decline (EOCD), which poses diagnostic and management challenges. The Cognitive and Neuroimaging for Neurodegenerative Disorders (CogNID) study aims to characterise clinical, cognitive, neuroimaging, and biomarker features across a diverse cohort of individuals with cognitive impairment, with a focus on diagnostic complexity, biomarker utility, and mortality.

**Methods:**

Out of 680 study participants within this prospective cohort enrolled from the real-world clinics within the National Health Service, who consented to take part in the study, we analysed data from 429. Individuals were recruited between December 2018 and November 2024 from the Memory Clinics, including the early-onset dementia service and associated services. Participants underwent structured cognitive assessments, neuroimaging (MRI/CT), and Cerebrospinal fluid (CSF) biomarker evaluation, where available. Diagnoses were made by multidisciplinary consensus. Group comparisons were conducted between early-onset (EOCD, <65 years) and late-onset cognitive decline (LOCD, ≥65 years).

**Results:**

Of the 429 participants, 349 (81.4%) had EOCD and 80 (18.6%) had LOCD. The mean age was 60.05 years, with no significant difference in sex or ethnicity across groups. Depression and anxiety were common (29.6%), as were cardiovascular risk factors. Lumbar punctures were more frequently performed in EOCD (p = 0.03), with 36.4% of tested participants demonstrating biomarker profiles consistent with Alzheimer’s disease (A+T+). Functional cognitive disorder (FCD) was more common in EOCD (22.3% vs. 5.0%, p < 0.001). Subgroup analysis revealed significantly lower ACE-III scores and higher pathological CSF findings in Alzheimer’s disease versus FCD. Mortality was higher in the LOCD group (11.3% vs. 4.6%, p = 0.03).

**Conclusion:**

The CogNID study highlights the clinical and diagnostic heterogeneity of individuals with cognitive impairment, particularly in younger adults. Incorporating neuroimaging and CSF biomarkers into routine clinical pathways enhances diagnostic precision and reveals distinct phenotypic profiles between EOCD and LOCD. These findings underscore the need for harmonised diagnostic protocols, broader biomarker accessibility, and inclusive recruitment strategies in dementia research and clinical services.

## Introduction

Early-onset Cognitive Decline (EOCD) poses a distinct clinical challenge, especially due to the diversity in underlying causes and the complexity in making an accurate diagnosis. Although it is estimated that 70,800 people in the UK have been diagnosed with early-onset dementia (1), this number is likely to be a significant underestimate. The true figure could be much higher, with a potential 19,000 undiagnosed cases in England alone (2). Dementia in adults under the age of 65 years can present differently to late-onset dementia, particularly regarding psychiatric and behavioural symptoms (3). This can lead to misdiagnosis, or a delay in reaching a EOCD diagnosis. A review of the 2019 English national memory service audit found that those under the age of 65 years were around 40% less likely to get a correct dementia diagnosis (4). Compounding the difficulty, EOCD accounts for just 119 per 100,000 cases (5) compared to functional disorders like schizophrenia, which presents at 1 per 100 cases (6).

In younger populations, the aetiological diversity is greater compared to older adults, where Alzheimer’s disease (AD) accounts for most dementia cases. In younger adults, AD represents less than half of diagnoses, with frontotemporal lobar degeneration (FTLD) and other rarer disorders contributing to over 30% of cases (7). In a review from Hendriks et al., the prevalence of AD in adults aged 30–64 years was estimated as 54.1 per 100,000 in the European population. Vascular dementia was estimated as 19.5 per 100,000 in the European population and FTLD was 2.9 per 100,000 (5).

EOCD has a profound impact not only on the individual but also on their family and wider social network. Individuals are often amid their working lives, and many face early job loss or forced retirement, leading to financial hardship and a loss of personal identity. Unlike those with late-onset dementia, individuals with EOCD may have dependent children or caregiving responsibilities, which places additional emotional and logistical burdens on families (8). These challenges highlight the urgency of early and accurate diagnosis, access to appropriate services, and the development of preventative strategies to reduce the wider impact of the disease (9).

There is a growing evidence base to support the role of modifiable risk factors in the development of dementia. In the Lancet Commission on dementia prevention, intervention and care, they estimated that 45% of dementia cases may be preventable by addressing the risk factors throughout the life span, such as smoking, alcohol consumption, and education level (10). This complexity, along with emerging concerns such as cognitive impairment related to COVID-19, highlights the need for robust, multifaceted diagnostic approaches.

Longitudinal studies provide fundamental information in understanding the diverse population living with dementia, and their diagnosis and care journey. These studies enable researchers to explore the biological, social, and environmental factors which affect individuals and better understand how these interact to affect cognitive decline (11). However, longitudinal data often collected through cohort studies is not readily available, and research has been limited in certain parts of the globe (11).

The UK National Health System represents a valuable source of clinical data, including longitudinal data from real-world clinical practice. Incorporating research models with ethically approved protocols and consents compliant with the General Data Protection Regulation (GDPR) would enable harmonised data collection and effective use of this resource. Considering recent advanced and multi-modal imaging and biomarker investigations, as well as emerging therapies, integrating research frameworks and ethically approved consent models into NHS data collection would be crucial to support the implementation of clinical guidelines, reduce healthcare burden, and centralise data collection. This would enable higher-impact research, enhance training, and ensure clinical studies are grounded in robust, meaningful real-world data.

### Neuropsychological assessment

Neuropsychological assessments are used routinely to provide objective evidence of cognitive difficulties (12). However, existing neuropsychological tools are often insufficient in the early and accurate diagnosis of dementia, especially in patients with EOCD (12). The UK National Institute for Health and Care Excellence has recommended applying a validated brief structured cognitive instrument such as the 10-point cognitive screener (10-CS) and the 6-item cognitive impairment test (6CIT) for initial screen assessment in non-specialist settings (13). The Addenbrooke’s Cognitive Examination III (ACE-III; 14) is often used in clinical settings to detect any change in cognition. The ACE-III has a maximum score of 100 and questions explore five cognitive domains; memory, attention, language, verbal fluency, and visuospatial function (14). Evidence suggests that the ACE-III is more sensitive that other assessments, such as the Mini Mental State Examination, in the detection of dementia (15). Although the ACE-III has become routinely used in the UK memory clinics, it is important to consider demographic variables and modifiable risk factors for dementia when interpreting scores (15).

While neuropsychological assessments are helpful in identifying any initial cognitive changes, their scores should not be considered alone when diagnosing dementia (12). Clinical assessments in isolation are inaccurate in 30-35% of cases (16). The incorporation of biomarkers and neuroimaging is essential for establishing a multidimensional diagnostic approach, improving the likelihood of accurate diagnosis and reducing the risk of misdiagnosis or under-diagnosis, particularly for patients with EOCD (12).

### Rationale

The aim of this study was twofold:

First, to standardise cognitive assessments, neuroimaging, and biomarker investigations for patients referred to neurology-led tertiary referral clinics. This initiative was designed to support regional memory clinics by implementing a harmonised protocol for cognitive evaluation, neuroimaging, and the use of biomarkers in accordance with NICE guidelines. This approach was particularly important during a period of clinical uncertainty regarding the indications or lack of a nationwide accessibility for Cerebral Spinal Fluid (CSF) biomarkers or Amyloid-PET imaging in routine UK clinical practice.

Secondly, to establish a research platform that would maximise the use of underutilised but costly investigations performed through the National Health Service. This platform aimed to generate real-world clinical data to inform demographic profiles, particularly of populations commonly under-represented in research. It also sought to leverage clinical and neuroimaging data for ongoing and future studies. In addition, individuals with mild cognitive impairment (MCI) were offered opportunities to participate in parallel and future research. For participants undergoing diagnostic lumbar puncture, the option to donate additional CSF samples for research was provided, enabling complementary studies on neurodegenerative biomarkers.

In this article, we introduce the Cognitive and Neuroimaging for Neurodegenerative Disorders (CogNID) Study, a longitudinal observational clinical study involving participants experiencing cognitive difficulties. We present the outline of the study, the measures we use, and the initial findings from the study’s six years of recruitment and data collection.

### Objectives

#### Primary objectives:

1. To identify cognitive and neuroimaging markers that can assist in the early diagnosis of neurodegenerative disorders. This includes assessing predictors of disease progression and identifying risk factors within diverse ethnic communities and across the lifespan, with a particular focus on younger patients.
2. To establish a diverse research platform that includes an EOCD alongside a control group, enabling further research into neurodegenerative disorders and providing a foundation for longitudinal studies that integrate clinical, imaging, and biomarker data.

#### Secondary objectives:

1. To evaluate the relationship between clinical features and neuroimaging findings, aiming to identify patterns that correlate with early markers of neurodegenerative disorders.
2. To track the progression of cognitive decline and corresponding neuroimaging changes over time, facilitating the identification of biomarkers associated with disease trajectory.
3. To identify risk factors for neurodegenerative conditions by incorporating a control group, allowing comparative analysis and examination of factors that may modify disease progression.
4. To share data with open-access repository datasets, integrating cognitive, neuroimaging, and biomarker data to enable broader research collaboration and facilitate secondary analyses.

## Materials and methods

### Ethics approval

This study has been reviewed and approved by the NHS East Midlands Derby Research Ethics Committee (18/EM/0292; IRAS: 250525). All amendments and protocol changes have been reviewed and authorised by Nottingham University Hospitals NHS Trust and the above ethics committee, before being implemented. The study has also been reviewed and approved by the Research & Innovation Department of Nottingham University Hospitals NHS Trust. The CogNID Study has been accepted by ClinicalTrials.gov (NCT03861884). All participants must provide written informed consent prior to any data collection.

### Study design and setting

Research activities for this cross-sectional and longitudinal observational cohort study were carried out by researchers and clinicians working in the Queen’s Medical Centre, Nottingham University Hospitals NHS Trust, and the University of Nottingham. All cognitive assessments were carried out in-person within Nottingham University Hospitals NHS Trust, and conducted by either a clinician, assistant psychologist, or trained research practitioners.

This study initially focused on recruiting EOCD who were primarily referred from primary or secondary care within the region. However, as often occurs in real-world clinical practice, patients from older age groups, cognitive consequences related to COVID-19, and those referred from other regions were also included. Accordingly, the study protocol was amended, and by November 2024, the cohort included 80 late-onset dementia cases (18.65%).

### Recruitment

Participants were recruited from memory clinics based at Nottingham University Hospitals NHS Trust, and Nottinghamshire Healthcare Foundation Trust. An Additional older age control group were recruited from Cognitive Disorders clinics at Nottingham University Hospitals NHS Trust. Patients attending these clinics have been referred by primary and secondary care services within the East Midlands region including Nottinghamshire, Derbyshire, Leicestershire, and Lincolnshire. Nottingham University Hospitals NHS Trust is also a tertiary referral centre for the cerebrospinal fluid (CSF) diagnostic process, and some participants were recruited via this pathway.

Eligible and interested patients received a Participant Information Sheet outlining the background and purpose of the study to read and were given the opportunity to ask questions and then complete and sign an Informed Consent Form. Some participants were co-recruited for a study involving blood tests and CSF examinations (MREC reference 08/H0408/167). Additional blood and CSF samples were donated for research purposes.

### Inclusion and exclusion criteria

All potential participants needed to have the ability and capacity to give consent prior to study enrolment. Capacity was based on the criteria defined by the Mental Capacity Act 2005, and any queries pertaining to an individual’s capacity were resolved through discussions with a medical professional.

#### Inclusion criteria

• Aged >18 years and ≤ 75 years. We modified it to Aged >18 to include older age for all eligible participants in 2024;

• Experiencing cognitive symptoms or cognitive impairment and screened and referred by the primary care team to our diagnostic dementia service;

• Pathological, genetic, or imaging biomarker evidence to suggest FTLD, AD or Vascular Cognitive Impairment (VCI);

OR

• A clinical diagnosis of one of the following which fall under the named conditions above:

o FTLD – Frontotemporal dementia; behavioural variant, semantic dementia, progressive non-fluent aphasia, progressive apraxia of speech; Progressive supranuclear palsy; Corticobasal degeneration; Poorly differentiated tauopathy with compound features of FTLD;

o AD – Amnestic (classic) Alzheimer’s disease; Posterior cortical atrophy; Logopenic or mixed primary progressive aphasia; Frontal variant Alzheimer’s disease, and Limbic-predominant Age-related TDP-43 Encephalopathy;

o Dementia with Lewy Body (DLB); Parkinson’s Disease Dementia (PDD);

o VCI – Hereditary vascular dementia; multi-infarct dementia; post-stroke dementia; subcortical ischaemic vascular disease; cerebral amyloid angiopathy spectrum;

o MCI secondary to any neurodegenerative or neurometabolic disorder such as neurodegeneration with brain iron accumulation(NBIA) or primary familial brain calcification;

o Functional cognitive disorder (FCD) – defined to be a condition without objective evidence of neurodegeneration. The affected individual reports cognitive difficulties but retains normal daily functioning abilities, with no detectable neurodegenerative biomarkers.

#### Exclusion criteria

• Unable to provide informed consent;
• Not speaking English before the age of five years; This criterion was removed to include any spoken language and ensure inclusivity of ethnic minorities in 2024;
• Presence of conditions such as learning disability, substance misuse, major depression, schizophrenia, or use of medications known to significantly impact the study participant’s cognition;
• Unable to sustain concentration for at least 30 minutes, in single sitting.

### Assessments

Comprehensive data collected across multiple domains for participants enrolled in the study (**Table 1**). Once consented, all participants underwent an ACE-III cognitive assessment during, or shortly after, their first visit to a clinic. If a participant has recently completed an ACE-III with an experienced clinical team member, a copy of this assessment was used instead. There were alternative cognitive assessments available within the clinic, if required. These included the Montreal Cognitive Assessment (MoCA; 17); the Birmingham Cognitive Screen (BCoS; 18); the Uniform Data Set (UDS; 19); and the Rowland Universal Dementia Assessment Scale (Rudas; 20). If low mood was identified during the clinic visit, it was assessed with the Hospital Anxiety and Depression Scale (HADS; 21). The Neuropsychiatric Inventory (NPI; 22) was used as an optional assessment, should the clinician wish to further explore the psychopathology of symptoms.

**Table 1:** Overview of clinical, cognitive, neuroimaging, and biomarker measures in the study cohort.

Category	Measures
Clinical Phenotyping	Detailed cognitive, neurological, neuropsychiatric assessments, CSF analysis Deep clinical phenotyping data based on the extensive clinical, neurocognitive, imaging and biological data collections and multidisciplinary discussions.
Cognitive Assessment	ACE-III - collected at baseline and at the time of follow-up to assess the trajectory of progression. In some cases, alternative methods of assessment are used including MoCA, BCoS, and UDSNB3.0
Psychometric	HADS NPI
Neuroimaging	3D or 2D MRI of the brain at 1.5T or 3T: T1-Weighted, T2-TSE, T2 FLAIR, Diffusion-Weighted, SWI/gradient echo sequences. If MRI was contraindicated: CT scan of the brain (small number of participants; n=10) FDG-PET CT of the head Functional MRI and MR spectroscopy (selected cases with aphasia features) Ultrahigh-Field MRI at 7T (selected cases with early Alzheimer’s disease)
CSF Biomarkers	CSF biomarkers: CSF Amyloidβ42/Amyloidβ40 ratio, total tau, 181-Phosphorylated-tau (The CSF Dementia Biomarker Service at the NHS Neuroimmunology and CSF Laboratory, University College London).
Genetic Testing	Genetic tests for early-onset dementia, FTLD, or dementia with strong family history such as R58 gene panel testing for adult-onset neurodegenerative disorders (https://panelapp.genomicsengland.co.uk/panels/474/).

ACE-III, Addenbrooke’s Cognitive Examination-III; MoCA, Montreal Cognitive Assessment; BCoS, Birmingham Cognitive Screen; UDSNB3.0, Uniform Data Set, version 3-Form B; HADS, Hospital Anxiety and Depression Scale; NPI, Neuropsychiatric Inventory; CSF, cerebrospinal fluid; FDG-PET, fluorodeoxyglucose positron emission tomography; MRI: magnetic resonance imaging; CT, computed tomography; SWI, susceptibility-weighted imaging; DWI, diffusion-weighted imaging; CSF, cerebrospinal fluid; FTLD, frontotemporal lobar degeneration.

Participants consented to share their clinical data, which includes pseudonymised images and clinical reports of 1.5T or 3T MRI, CT brain, FDG-PET CT brain scans, Dopamine Transporter (DaT) Scans, the results of diagnostic lumbar punctures, and biochemical blood tests. These tests include cell count, renal and liver function tests, blood glucose, thyroid function, vitamin B12, and folate levels, as well as, in selected cases, autoimmune encephalitis antibodies and infection screens.

Imaging protocol for the brain MRI included 3D structural T1-Weighted, T2-Weighted, FLAIR, Diffusion-weighted, and gradient echo or susceptibility images at 3T or 1.5T MRI. Participants may consent to undergo advanced neuroimaging modalities, such as MR spectroscopy, functional MRI or ultra-high-field MRI at 7T (23, 24). Additional advanced neuroimaging was undertaken within Nottingham University Hospitals NHS Trust or the Sir Peter Mansfield Imaging Centre, University of Nottingham.

Diagnostic lumbar puncture provides information on CSF biomarkers, including the Amyloid β1-42 (Aβ42), Amyloid β1-40 (Aβ40), total tau, and 181-phosphorylated tau (25). However, in the initial stages of the study, the national laboratory testing for CSF biomarkers only reported Aβ42 without Aβ40. As a result, this Aβ42/Aβ40 ratio was unavailable for selected cases.

Patients were reviewed by the Early-onset Dementia multidisciplinary team including psychiatrists, neurologists, neuroradiologists, psychologists, dementia nurses and occupational therapists. Home visits were also offered when required to understand functional status, home environment and social circumstances. Monthly meetings were held to discuss complex cases and team approach. Evidence has shown that a collaborative interdisciplinary approach improves patient outcomes (26).

### Follow-up assessments

Participants were followed up at 6 and 12 months, then annually, with optional repeat imaging and psychometric assessments. Follow-ups may be conducted in-person, via telephone, or remotely. Repeat MRI scans arranged one year after recruitment for participants who consent.

### Statistical methods

Descriptive statistics were used to summarise demographic, cognitive, and biomarker data. Continuous variables were assessed for normality and were presented as means with standard deviations (SD) for normally distributed data, or as medians with interquartile ranges (IQR) for non-normally distributed data, as appropriate. Statistical analyses were conducted to compare demographic and clinical characteristics between the early-onset and late-onset dementia groups. Independent samples t-tests were used for continuous variables that met the assumption of normality, while the Mann–Whitney U test was applied to non-normally distributed data. Categorical variables were assessed using chi-square tests; where expected cell counts were less than five, Fisher’s exact tests were employed. A p-value of less than 0.05 was considered statistically significant. All analyses were performed using Python, with the scipy.stats libraries.

## Results

### Recruitment and demographics

**Figure 1:**
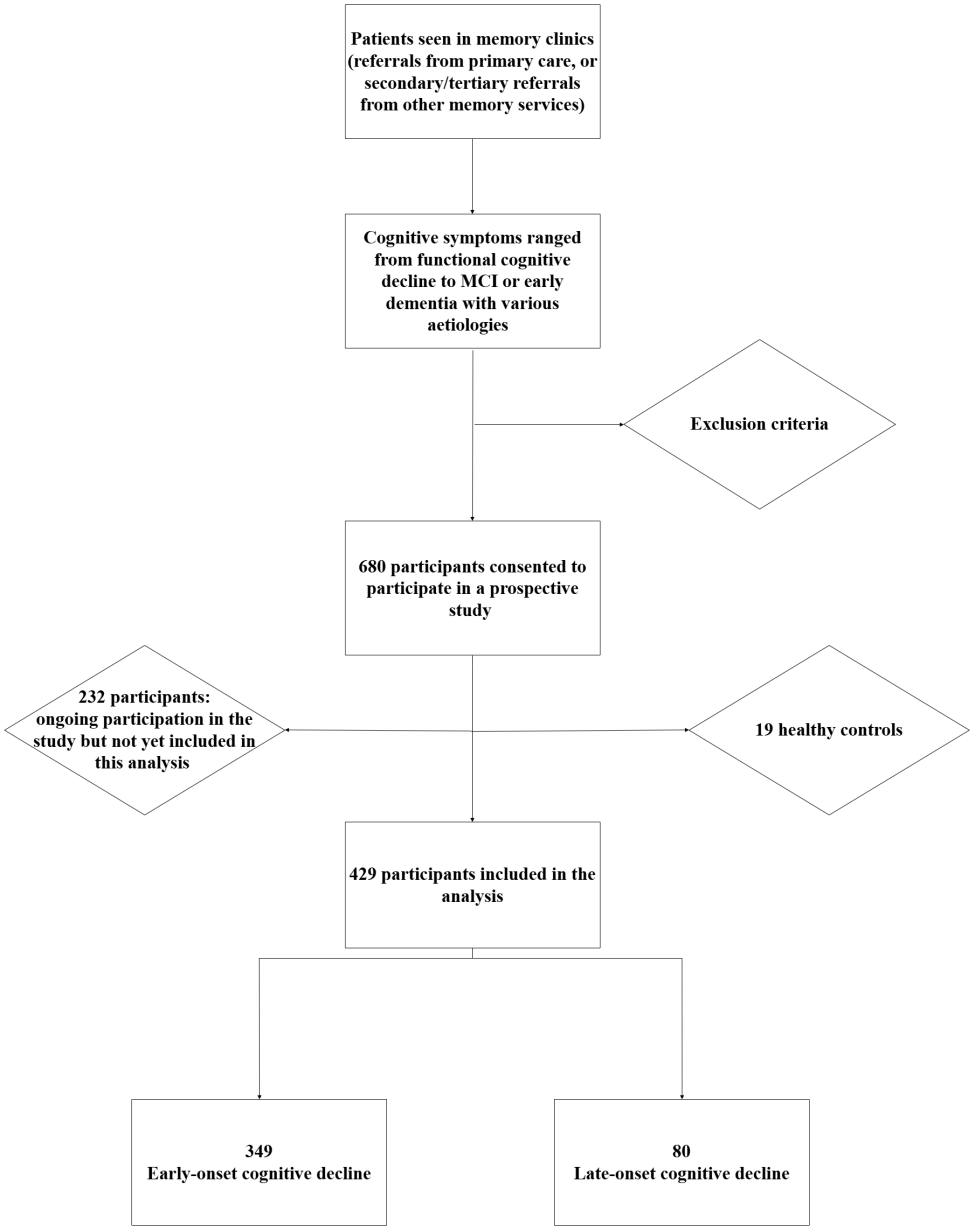
Recruitment flowchart.

Between December 2018 and April 2025, a total of 680 participants, who attended our memory clinics, consented to take part in the clinical study for research in dementia. Of these, the data until November 2024 were analysed in this report. After excluding 19 healthy controls, the final analytical cohort comprised 429 individuals with a clinical diagnosis of dementia (see **Figure 1**). The mean age of participants was 60.05 years (range 18–84), with a slight male predominance (54.6%). The majority were of Caucasian ethnicity (63.9%). Regarding lifestyle factors, 5.1% of participants were current smokers, 26.3% reported current alcohol consumption, and 2.3% reported excessive alcohol use (see **Table 2**).

**Table 2:** Demographic and clinical characteristics of participants with early-onset and late-onset cognitive decline.

Demographics	Total N=429	EOCD N= 349	LOCD N=80	P
Age, mean ±SD	60.05±11.08	57.00± 9.82	73.35±4.68	<0.001*
Male, n(%)	234 (54.6)	190 (54.4)	44 (55.0)	1.00
Ethnicity				
Caucasian, n(%)	274 (63.9)	219 (62.8)	55 (68.8)	0.38
Smoking				
Current smoker, n(%)	22 (5.1)	22 (6.3)	0 (0)	0.02*
Alcohol				
Current drinker, n(%) History of alcohol excess, n(%)	113 (26.3) 10 (2.3)	92 (26.4) 10 (2.9)	21 (26.3) 0 (0)	1.00 0.22
Comorbidities				
Number of comorbidities Head injury, n(%) Domestic repetitive head injury, n(%) Depression/Anxiety, n(%) T2DM, n(%) Hypertension, n(%) Cardiovascular disease, n(%) History of Stroke/TIA, n(%)	0.73(0.96) 13 (3.0) 4 (0.9) 127 (29.6) 42 (9.8) 68 (15.9) 32 (7.5) 27 (6.3)	0.75(0.95) 10(2.9) 4(1.1) 109(31.2) 36 (10.3) 56 (16.0) 26(7.4) 21(6.0)	0.64(1.00) 3 (3.8) 0(0) 18 (22.5) 6(7.5) 12 (15.0) 6(7.5) 6 (7.5)	0.34 0.72 1.00 0.16 0.58 0.95 1.00 0.81

EOCD, Early Onset Cognitive Decline; LOCD, Late Onset Cognitive Decline; SD: standard deviation; T2DM, Type 2 diabetes mellitus; TIA, Transient ischaemic attack;* indicate statistically significant differences at *p* < 0.05.

Out of the recruited 429 individuals, 349 (81.4%) had EOCD and 80 (18.6%) had LOCD. The mean age in the EOCD was 57.00 ± 9.82 years, whereas the LOCD was 73.35 ± 4.68 years (p < 0.001). The proportion of males was similar between groups (54.4% early-onset vs. 55% late-onset; p = 1.0). Caucasian ethnicity was the most common across both groups, with no significant difference between EOCD (62.8%) and LOCD (68.8%; p = 0.38). Current smoking was significantly more prevalent in the EOCD group (6.3%) compared to none in the LOCD group (p = 0.02). There were no significant differences in alcohol consumption or history of alcohol excess between groups.

The demographic data revealed notable patterns of comorbidities among the 429 individuals in the cohort, emphasizing the multifactorial health challenges present in the population. Depression and anxiety were the most prevalent comorbid conditions, affecting 127 individuals (29.6%), which aligns with their frequent association with chronic and neurological conditions. Hypertension, a common risk factor for cardiovascular and neurological diseases, was present in 68 individuals (15.9%), while Type 2 Diabetes Mellitus (T2DM) affected 42 individuals (9.8%). Cardiovascular disease was reported in 32 individuals (7.5%), reflecting a significant burden of systemic health issues. Additionally, 27 individuals (6.3%) had a history of stroke or TIA, indicating a notable prevalence of cerebrovascular complications. Less common comorbidities included head injury (13 individuals, 3.0%) and a history of domestic abuse (4 individuals, 0.9%). These findings highlight the importance of addressing mental health, metabolic disorders, and cardiovascular risks in managing this population comprehensively (10).

Co-existing medical and mental health conditions (i.e. rates of depression/anxiety, T2DM, hypertension, cardiovascular disease, and history of stroke/TIA) were comparable between EOCD and LOCD groups, with no statistically significant differences observed (**Table 2**).

### Neurocognitive assessment, neuroimage and biomarkers

The median total ACE-III score among all participants was 73 (**Table 3**). A threshold score of <82 was used to identify possible MCI, as suggested by Hsieh et al. (14), and was observed in 72.2% of the cohort. The median sub-scores were as follows: Attention – 14, Memory – 15, Fluency – 8, Language – 23, and Visuospatial skills – 14. Age-specific cut-offs will be considered in subsequent analyses, in line with recommendations by previous study (27).

**Table 3:** Details of cognitive assessments, neuroimaging investigations and biomarkers among different neurodegenerative disorders within the study population.

Measurements	Total N=429	EOCD N=349	LOCD N=80	P
Initial ACE-III				
Total, median (IQR) Attention, median (IQR) Memory, median (IQR) Fluency, median (IQR) Language, median (IQR) Visuospatial, median (IQR) Total ≤82, n(%)	73 (61-84) 14 (12-17) 15 (10-20) 8 (5-10) 23 (20-25) 14 (11-15) 252 (72.2)	73 (59.8-84.3) 14 (12-17) 15 (10-26) 8 (5-10) 23 (19-25) 14 (10-15) 206 (70.6)	71 (62-81) 14.5 (12-17) 15 (10-20.3) 8 (5-10) 24 (21-25) 14 (12-15) 46 (80.7)	0.88 0.82 0.90 0.76 0.48 0.36 0.16
CT or MRI performed, n(%)	396 (92.3)	322 (92.3)	75 (93.8)	0.83
MRI performed, n(%)	386 (90.0)	317 (90.8)	69 (86.3)	0.31
LP performed, n(%)	150 (35.0)	131 (37.5)	19 (23.8)	0.03*
CSF t-tau >595, n(%)	46 (30.7)	38 (29.0)	8 (42.1)	0.37
CSF p-tau-181 >57, n(%)	65 (44.2)	53 (41.4)	12 (63.2)	0.13
CSF Aβ42 abnormal levels**<627, n(%)	105 (70.0)	88 (67.2)	17 (89.5)	0.09
CSF Aβ42/Aβ40 ratio <0.065, n(%)	55 (49.5)	45 (45.5)	10 (83.3)	0.03*
AD Pathology A+T+ A+T- A-T+ A-T-	40 (36.4) 15 (13.6) 7 (6.4) 48 (43.6)	33 (33.7) 12 (12.2) 6 (6.1) 47 (48.0)	7 (58.3) 3 (25) 1 (8.3) 1 (8.3)	0.07
Mortality, n(%)	25 (5.8)	16 (4.6)	9 (11.3)	0.03*
Common Diagnoses, n(%) -AD -FCD, Multifactorial, Mental health, FND -FTDL -VCI -COVID-related cognitive syndromes -PDD -DLB -Co-pathology -Others	114 (26.6) 82 (19.1) 34 (7.9) 30 (7.0) 24 (5.6) 12 (2.8) 12 (2.8) 22 (5.1) 99 (23.1)	89 (25.5) 78 (22.3) 24 (6.9) 23 (6.6) 19 (5.4) 8 (2.3) 6 (1.7) 16 (4.6) 86 (24.6)	25 (31.3) 4 (5.0) 10 (12.5) 7 (8.8) 5 (6.3) 4 (5) 6 (7.5) 6 (7.5) 13 (16.3)	<0.001*

EOCD: Early Onset Cognitive Decline; LOCD: Late Onset Cognitive Decline; ACE-III: Addenbrooke’s Cognitive Examination III; IQR: the interquartile range; LP: lumbar puncture; CSF: cerebrospinal fluid; Aβ: amyloid-beta; t-tau: total tau; p-tau: phosphorylated tau; AD: Alzheimer’s disease; A+T+: evidence of Amyloidβ (A+) and tau (T+) based on the ATN criteria; FTLD: frontotemporal lobar degeneration; VCI: vascular cognitive impairment; DLB: dementia with Lewy body; FCD: functional cognitive disorder; FND: functional neurological disorder; PPD: Parkinson’s Disease Dementia. CSF biomarker units: Amyloidβ1-42 (Aβ42), total tau (t-tau), and 181-phosphorylated-tau (p-tau-181) concentrations are reported in pg/mL. *statistically significant. **The reference laboratory defined CSF Aβ42 abnormality as <627 pg/mL in 2018 and 2019 (n=39 study participants), when Aβ40 was not measured; it now reports both Aβ42 and Aβ40 concentrations (pg/mL) for NHS referrals.

92.3% participants had at least one form of neuroimages performed including CT or MRI head. MRI head scans were performed in 386 participants (90.0%). CSF analysis was completed in 150 participants (35.0%). Among those with available CSF data, elevated total tau (t-tau >595 pg/ml) was present in 30.7%, elevated phosphorylated tau (p-tau-181 >57 pg/ml) in 44.2%, reduced Aβ42 (<627 pg/ml) in 70.0%, and a reduced Aβ42/Aβ40 ratio (<0.065) in 49.5%. An additional 91 participants with available CSF samples were recruited during the manuscript preparation phase.

Biomarker confirmed AD was defined as presents with amyloidosis and tauopathy evident with reduced Aβ42/Aβ40 and increased p-tau-181 protein in CSF (28). 150 participants had diagnostic lumbar puncture performed. However, Aβ40 was not tested in cases initially due to test accessibility at the reference laboratory. Out of 110 participants that had both p-tau-181 and Aβ42/Aβ40, 40 cases (36.4%) fulfil these criteria with A+T+. Fifteen participants had CSF amyloidosis without tauopathy, while 7 cases had CSF tauopathy without amyloidosis.

Cognitive performance, as measured by ACE-III total and subdomain scores, did not significantly differ between EOCD and LOCD groups. MRI was performed frequently and comparably across both groups. However, lumbar punctures were significantly more common in the EOCD (p = 0.03), potentially reflecting greater diagnostic complexity or a wider differential diagnosis in younger individuals. CSF biomarker profiles revealed broadly similar levels of t-tau and p-tau-181 between groups. Although a higher proportion of LOCD participants exhibited low Aβ42 levels, this did not reach statistical significance (p = 0.09). In contrast, the proportion of participants with a pathological Aβ42/Aβ40 ratio (<0.065) was significantly greater in the LOCD group, suggesting a higher burden of Alzheimer’s pathology in older individuals.

### Diagnoses

The wide spectrum of diagnoses has been observed in this study, suggesting the complexity of differential diagnosis in dementia service (**Table 3**). Diagnosis distribution was significantly different between EOCD and LOCD groups (p<0.001). AD was the most prevalent diagnosis across the cohort (26.6%), with a higher frequency in the late-onset group (31.3%) compared to early-onset cases (25.5%). FCD and psychiatric causes, including non-neurodegenerative multifactorial, psychiatric, and functional presentations, were the second most common overall (19.1%) and significantly more frequent in early-onset cases (22.3%) than in late-onset (5.0%). FTLD, VCI, and COVID-related cognitive syndromes were present across both groups with similar distribution. PDD and DLB were more commonly diagnosed in the late-onset group (5.0% and 7.5%, respectively), relative to early-onset (2.3% and 1.7%). Co-pathology, evidenced by biomarkers, was also more prevalent among late-onset cases, while other diagnoses were observed more frequently in EOCD group. The two most common combinations were AD + FTLD (n=7) and AD + DLB (n=3). Overall, there was a statistically significant difference in diagnostic distribution between early- and late-onset dementia groups (p < 0.001), reflecting distinct clinical profiles based on age of onset. During the longitudinal follow-up, mortality was higher in the LOCD group (11.3% vs. 4.6% in EOCD, p=0.03), suggesting a poorer prognosis despite similar cognitive impairment severity and comorbidity burden.

### Alzheimer’s disease and functional cognitive decline

In this sub-analysis comparing patients diagnosed with AD (N=114) and FCD (N=55), several key demographic and clinical differences were found (**Table 4**). Patients with FCD were significantly younger than those with AD (mean age 58.44 vs. 63.27 years; p < 0.001). There were no significant differences in sex distribution, ethnicity, smoking status, or alcohol consumption between groups. MRI brain was performed at similar rates, as was CSF biomarker testing. There was no mortality in the FCD group, compared with a 6.1% mortality rate in the AD group).

**Table 4:** Demographic, cognitive, and CSF profiles of the two most common diagnoses among the study population.

Diagnosis	AD N=114 (26.6%)	FCD N=55 (12.8%)	P
Age, mean ±SD	63.27±7.20	58.44±6.46	<0.001 *
Early onset, n(%)	89(78.1)	52(94.5)	0.01*
Male, n(%)	56(49.1)	30(54.5)	0.62
Caucasian, n(%)	68(59.6)	40(72.7)	0.14
Current smoker, n(%)	6(5.3)	4(7.3)	0.73
Alcohol			
Current drinker, n(%) History of alcohol excess, n(%)	35(30.7) 2(1.8)	16(29.1) 1(1.8)	0.97 1.00
Initial ACE-III			
Total ≤82, n(%) Total, median (IQR) Attention, median (IQR) Memory, median (IQR) Fluency, median (IQR) Language, median (IQR) Visuospatial, median (IQR)	81(84.4) 63(53-76.5) 12(9-15) 11(9-16) 7(4-9) 22(19-24) 11(8-15)	28(60.9) 77.5(70-86) 16(12-17) 17(14-20) 8(5-11) 24(22-25) 15(13-15)	<0.05* <0.001* 0.001* <0.001* 0.03* 0.02* 0.001*
MRI performed, n(%)	103(90.4)	52(94.5)	0.55
CSF biomarker study, n(%)	70(61.4)	38(69.1)	0.42
CSF			
CSF t-tau >595, n(%) CSF p-tau-181 >57, n(%) CSF Aβ42 <627, n(%) CSF Aβ42/40 <0.065, n(%)	40(57.1) 54(77.1) 67(95.7) 46(92.0)	1(2.6) 3(7.9) 15(39.5) 0(0)	<0.001* <0.001* <0.001* <0.001*
Mortality, n(%)	7(6.1)	0(0)	0.10

AD, Alzheimer’s disease; FCD, functional cognitive disorder; SD, standard deviation; ACE-III, Addenbrooke’s Cognitive Examination III; IQR, the interquartile range; CSF, cerebrospinal fluid; Amyloidβ1-42 (Aβ42), total tau (t-tau), and 181-phosphorylated-tau (p-tau-181) concentrations are reported in pg/mL. Aβ42/Aβ40 is a unit-less ratio; CSF biomarker units: Aβ42, t-tau, and p-tau-181 concentrations are reported in pg/mL. Aβ42/40 is a unitless ratio. *statistically significant.

At initial assessment, participants with AD showed significantly lower cognitive performance across all domains of the ACE-III compared to those with FCD. A higher proportion of AD scored below the diagnostic threshold of 82 (84.4% vs. 60.9%, *p* < 0.05). The median total ACE-III score was notably lower in AD (63 [IQR 53–76.5]) than in the FCD group (77.5 [IQR 70–86], *p* < 0.001). Domain-specific analysis revealed significantly reduced scores in attention, memory, and visuospatial functions (all *p* ≤ 0.001), with additional but more modest differences in fluency (*p*=0.03) and language (*p*=0.02). These results suggest a broader and more severe cognitive profile at presentation among individuals with AD.

CSF biomarker profiles showed marked differences between AD and FCD participants. Abnormal levels of total tau (>595 pg/ml) were observed in 57.1% of AD participants, compared to only 2.6% in the FCD group (*p* < 0.001). Similarly, elevated phosphorylated tau (p-tau-181 >57 pg/ml) was present in 77.1% of AD cases versus 7.9% in the FCD group (*p* < 0.001). Reduced Aβ42/Aβ40 ratio (<0.065), indicative of A+ pathology, was detected in 92% of participants with AD, compared to none in the participants with FCD (*p* < 0.001).

### Mortality

During the longitudinal follow-up, 25 participants died (see **Table 5**). The mean age at death was 65.28±11.22 years, with the majority being male (72%) and 48% identifying as Caucasian. The mean duration of follow-up prior to death was 2.40±1.53 years. Most individuals (64%) had EOCD. AD (n=7) and FTLD (n=5) were the most common primary diagnoses, followed by DLB (n=3), VCI (n=2), corticobasal degeneration (n=1), PDD (n=1), autoimmune encephalitis (n=1), and epilepsy (n=1). Additionally, four individuals had co-pathologies. These data highlight the clinical and pathological diversity of neurodegenerative disorders associated with mortality in this cohort.

**Table 5:** Demographic, diagnostic, and clinical characteristics of deceased participants in the study cohort.

Mortality	N=25
Age of Death, mean ±SD	65.28±11.22
Male, n(%)	18(72)
Caucasian, n(%)	12(48)
Diagnosis, n (%)	
AD FTLD DLB VCI CBD PDD AIE Epilepsy Co-pathology (AD+FTLD, AD+DLB, CBD+PSP, FTLD+PSP)	7(28) 5(20) 3(12) 2(8) 1(4) 1(4) 1(4) 1(4) 4(16)
Duration in the study (year), mean ± SD	2.40±1.53
EOCD, n(%)	16(64)

SD, standard deviation; AD, Alzheimer’s disease; FTLD, frontotemporal lobar degeneration; VCI, vascular cognitive impairment; DLB, dementia with Lewy body; CBD, corticobasal degeneration; PDD, Parkinson’s Disease Dementia; AIE, Autoimmune Encephalitis; PSP, Progressive Supranuclear Palsy, EOCD, Early Onset Cognitive Decline.

## Discussion

### Overview

As defined as the onset of dementia symptoms before the age of 65 years, early-onset dementia accounts for less than 10% of all dementia. However, the disease burden has experienced an upward trend as global age standardised prevalence and incidence of early-onset dementia increased from 93.39 and 16.24 per 100,000 persons in 1990 to 96.09 and 17.16 per 100,000 persons in 2021 (29). As most previous studies were focused on the late-onset dementia, developing a clinical cohort of younger individuals with cognitive impairment provides a comprehensive overview of their demographic, clinical, and biomarker profiles. Memory services/clinics vary significantly in the UK. This study also gives an example of memory services.

The CogNID Study aims to explore and better understand the role of cognitive and neuroimaging factors in the early diagnosis or neurodegenerative conditions. Our initial data shows the wide range of age and diagnoses which attend the memory clinics locally. The detailed analyses offer insights into the spectrum of cognitive impairment, the prevalence of co-existing medical conditions, and the diagnostic yield of neuroimaging and CSF biomarkers in a heterogeneous clinical population.

This cohort predominantly included individuals with early-onset cognitive symptoms (81.4% EOCD vs. 18.6% LOCD), with a mean age of 60.05 years. Both groups shared a similar male predominance and ethnic distribution. The percentage of Caucasian participants was higher than the local census, given the protocol-driven exclusion criteria of ‘Not speaking English before the age of five years’ before 2024. Cultural differences may influence help-seeking behaviour (30), potentially resulting in an underrepresentation of certain groups within our sample, and across our clinic patient lists. Our cohort includes approximately 8% participants from ethnic minority backgrounds, while 28% have not disclosed their ethnicity. This highlights the challenges in capturing a fully representative sample. Although we amended the protocol inclusion criteria to remove being able to speak English before the age of five years, this was done at the end of the recruitment window included in this paper. Since the protocol amendment, 21.5% of newly recruited participants have been from ethnic minority groups, reflecting increased diversity in our study cohort. Therefore, it is likely that, in the early years of the CogNID study, opportunities to recruit participants from minority ethnic backgrounds were missed. Factors such as ethnicity, diversity, educational level, and illiteracy are important to consider. Evidence suggests that individuals from ethnic minority groups are less likely to attend clinics (31) and participate in research studies (32).

### Mental health, cardiovascular risk, and modifiable factors in dementia

Notably, the high prevalence of depression and anxiety in our cohort (29.6%) shows the complex interplay between neuropsychiatric symptoms and systemic comorbidities in individuals with cognitive impairment. Depression and anxiety are recognised both as early manifestations of neurodegenerative disease and as independent risk factors for progression to dementia. Longitudinal studies have shown that depression in MCI is associated with distinct clinical trajectories, with higher conversion rates to AD and VCI (33, 34). Moreover, meta-analyses, such as Yang et al. (35) using UK Biobank data, report that depression increases the risk of developing dementia by approximately 1.5-fold. Anxiety has also been independently linked to the later development of AD, suggesting that early neuropsychiatric screening may offer opportunities for earlier intervention (36).

Beyond mental health, cardiovascular risk factors were also prevalent, with hypertension affecting 15.9% and T2DM affecting 9.8% of participants. These findings align with epidemiological data from the Framingham Heart Study and others, which have demonstrated strong associations between midlife hypertension, diabetes, and later cognitive decline (37, 38). T2DM is associated with increased risk of both VCI and AD, with systematic reviews confirming the elevated prevalence of MCI among diabetic populations (38, 39).

Lifestyle factors also play a key role. Smoking has been consistently identified as a modifiable risk factor for both the development and progression of dementia. In younger populations, particularly, smoking may contribute to earlier onset through mechanisms such as vascular injury, oxidative stress, and neuroinflammation. Given the significantly higher prevalence of current smoking among early-onset dementia cases in our cohort (6.3% vs. 0% in late-onset), memory clinics and brain health services should actively promote smoking cessation as a primary prevention strategy (40, 41).

Overall, these findings reinforce the importance of recognising and addressing modifiable health behaviours, cardiovascular risks, and mental health symptoms as part of a comprehensive, multidisciplinary approach to dementia diagnosis and management.

### Neurocognitive and biomarker assessment

Cognitive evaluation using the ACE-III revealed that a substantial majority (72.2%) of patients scored below the established threshold. These deficits were similarly distributed between EOCD and LOCD groups, although these have not been age-adjusted.

The higher utilisation of lumbar puncture in the EOCD group (37.5% vs. 23.8%, p = 0.03) suggests that clinicians may be more inclined to employ advanced diagnostic tool when faced with atypical or diagnostically challenging presentations in younger patients. The high prevalence of abnormal Aβ42/Aβ40 ratio (49.5%) and tau (t-tau 30.7% and p-tau-181 44.2%) profiles in our cohort reinforces the utility of CSF studies, particularly in cases where traditional imaging modalities may be inconclusive.

In addition, ATN biomarker-based classification offers opportunities for more precise treatment selection and prognosis prediction. Stratifying patients based on their Aβ status (A+) and CSF tau status (T+) allows for an understanding of disease stage (42). For instance, individuals with an amnestic cognitive phenotype who are classified as A+T−, indicating abnormal Aβ but normal tau, are often considered to be in a preclinical or early prodromal stage of AD. This group may remain clinically stable for a longer period compared to those with both Aβ and tau abnormalities (A+T+), who are more likely to experience faster cognitive decline and progression to dementia (43). A recent study of CSF biomarkers suggested that a model disease staging using a combination of CSF biomarkers can be used to predict prognosis (44). Thus, the integration of CSF biomarker profiles into clinical practice not only enhances diagnostic accuracy but also supports a personalised medicine approach, guiding therapeutic decisions and informing patients about their likely disease course.

### Subgroup study: alzheimer’s disease and functional cognitive disorder

In the subgroup analysis, AD patients were older and exhibited more pronounced cognitive deficits than FCD groups. Despite similar rates of brain image and CSF biomarker testing between the groups, there was a marked difference in CSF biomarker abnormality as well as mortality. A retrospective study has shown that FCD patients had a lower age at present, higher MMSE scores and Geriatric depression scale scores compared with neurodegenerative disorders (45). FCD has been reported as a common diagnosis in memory clinics with a quarter of patients receiving diagnoses of FCD from memory clinics associated with psychogenic symptoms in a systematic review (46).

### Clinical and diagnostic considerations

The NICE guidelines (47), which are followed in our practice, recommend cognitive, radiological and CSF assessments. However, as most participants in our cohort are younger, with atypical presentations rarely observed in older groups, there are currently no specific guidelines tailored to this population. Further research into the diagnosis of those with EOCD is therefore needed to develop evidence-based guidelines that comprehensively address their diagnostic requirements.

The integration of neuropsychological testing, advanced neuroimaging, and CSF biomarker analysis provides a multidimensional approach to the diagnosis of cognitive disorders, particularly in a population where early-onset presentations may pose unique diagnostic challenges. The increased diagnostic yield in EOCD cases using lumbar puncture may reflect not only the diagnostic complexity in this subgroup but also a broader differential diagnosis considered by clinicians in younger patients.

Interestingly, co-pathologies have been observed in our cohort, underscoring the role of multiple pathological processes in the clinical manifestation of cognitive symptoms (42). In the Brains for Dementia Research study, histological evidence revealed that many participants exhibited co-pathologies, such as amyloidosis alongside TDP-43 proteinopathy (48). Emerging plasma biomarkers such as measuring glial fibrillary acidic protein (GFAP) and utilising alpha-synuclein (αSyn) seed amplification assay as well as neuroimaging techniques, including MRI and FDG-PET, may aid in detecting additional pathologies implicated in disease progression, such as vascular brain injury, neuroinflammation, and α-Syn (42). The presence of co-pathologies is often associated with more rapid cognitive decline and a poorer prognosis (49).

Plasma biomarkers such as phosphorylated tau-217 (p-tau-217) have quickly moved into clinical practice and trial eligibility criteria in countries including the United States, where both immunoassay and mass-spectrometry methods report high diagnostic accuracy for brain Aβ and tau pathology (50). In the United Kingdom, a large-scale clinical trial has been launched to evaluate the accuracy, feasibility, and scalability of plasma p-tau-217 and other plasma assays in diverse real-world settings.

### Limitations

The sample for this study has limited generalisability, as all participants were recruited from memory services within the East Midlands and restricted to those referred to a tertiary, neurology-led service. This recruitment pathway depends on patients receiving a diagnosis and accessing these services. In addition, individuals with known causes of cognitive impairment (e.g., following major stroke, traumatic brain injury, or substance misuse) were excluded, which may reduce the representation of co-pathologies commonly associated with dementia. Smoking and alcohol use within the cohort were likely underreported, possibly due to clinicians not routinely enquiring about these factors or patients feeling uncomfortable disclosing such information (51). As excessive alcohol consumption and smoking are recognised the risk factors for dementia (40), these findings suggest that clinical assessments should more consistently and systematically address lifestyle habits.

Representation of ethnic minority groups within the cohort was also limited, despite evidence that these populations have a higher risk of dementia, later presentation, and higher mortality rates (52–54). Furthermore, dementia presentations may vary between ethnic groups. For example, South Asian individuals have a higher prevalence of hypertension, obesity, diabetes, low high-density lipoprotein and sleep disorders compared to White individuals (55). Caribbean and African individuals share a high rate of cardiovascular disease (including hypertension and diabetes) with South Asian groups: however, additional socioeconomic, environmental, and genetic factors (such as ABCA7 gene) also contribute to risk (56).

Cognitive assessments in dementia are further influenced by differences in education, language and social background, which can bias diagnostic accuracy (57). To address this, since November 2024, our protocol has been revised to enable the inclusion of non-English-speaking participants by offering translated study materials, for example Arabic, Urdu, Polish and Gujarat ACE-III and interpreter support where required. Within our service, the Rowland Universal Dementia Assessment Scale (RUDAS) (20) has been implemented to help mitigate language and educational biases. Other culturally sensitive cognitive tools such as the Visual Cognitive Assessment Test and the Kimberley Indigenous Cognitive Assessment have been shown to be superior to the Mini-Mental State Examination for assessing dementia in ethnic minority populations (58) and could complement RUDAS in future research studies.

### Impact and future directions

The participants in this study, drawn from real-world clinical practice, presented with a spectrum of conditions, including subjective cognitive concerns, early-stage neurodegeneration, and mixed neurodegenerative pathologies (**Table 3**). Our clinical service uses a tiered assessment process (vetting system) and multidisciplinary reviews, incorporating assessments, discussions, and specialist input to guide appropriate investigations, stratification and follow-up. A standardised referral checklist for cognitive complaints in primary care, along with the use of harmonised objective cognitive tools (currently ACE-R or ACE-III in the UK practice, though potentially subject to future refinement), could streamline the referral pathway.

To ensure the long-term success and sustainability of this study, a structured implementation and evaluation strategy will be essential in future phases. This should involve close engagement with clinicians, commissioners, and patient representatives to co-design delivery models that are responsive to real-world service pressures. In parallel, the development of training materials and support frameworks for healthcare professionals will be important to facilitate effective implementation. Integration with existing digital health infrastructure and NHS systems is also anticipated to support routine data capture in clinical settings.

A future priority is the establishment of a dementia registry to promote consistent data collection across participating sites and to foster cross-centre learning. Such a shared framework will enhance diagnostic reliability and enable the longitudinal tracking of disease trajectories.

Our collaboration with Dementia Platform UK (DPUK) (https://www.dementiasplatform.uk/) facilitates open access sharing of de-identified imaging and clinical data, with strict adherence to data protection protocols, including double pseudonymisation and robust confidentiality procedures. This approach is designed to support broader research collaboration and accelerate discovery across the dementia research community.

Finally, the study provides as a valuable platform for medical education, research training, supporting the development of the next generation of clinicians and scientists with real-world exposure to dementia care and translational research.

## Conclusions

Longitudinal data are essential for providing deeper insights into the prevalence and characteristics of cognitive disorders within tertiary referral centres. Our centre serves a diverse population, offering valuable opportunities to implement and evaluate diagnostic, preventive, and therapeutic strategies over time. The CogNID study highlights the heterogeneity of cognitive presentations in a young-to-midlife population and underscores the importance of a multidisciplinary diagnostic approach, incorporating cognitive assessments, neuroimaging, and biomarker profiling.

Although recruitment limitations—such as regional sampling and referral bias—must be acknowledged, the growing size and diversity of our cohort strengthen its future research potential.

As this is an ongoing study, with the number of participants now exceeding 600, future follow-up data will allow us to investigate longitudinal outcomes, explore predictors of disease progression, and assess the impact of interventions. Ultimately, these efforts aim to improve early diagnosis, personalise treatment pathways, and contribute to the development of specific clinical guidelines for early-onset dementia.

## Data Availability

The raw data supporting the conclusions of this article will be made available by the authors, without undue reservation.
